# Addressing responsibility in innovation processes for sustainability: Lessons for responsible management of sustainable innovation form a systematic literature review

**DOI:** 10.3389/frma.2022.1057378

**Published:** 2022-11-24

**Authors:** Delia Mangelkramer

**Affiliations:** Chair of Innovation Management, Freie Universität Berlin, Berlin, Germany

**Keywords:** Responsible Research and Innovation (RRI), sustainable innovation, innovation process, system change, system transformation, systematic literature review

## Abstract

Analyzing the impact of a sustainability agenda in research and innovation on system transition is a critical research topic. This literature stream aims to examine how research and innovation can deal with wicked-problems at a dynamic system level to create more sustainable future systems. However, this study addresses two main issues in the current sustainability transition literature. First, the literature to date offers little insight into concrete implications for the management of innovation processes at the organizational level. Second, sustainability is often addressed as *per se* desirable. While the concept of Sustainable Innovation (SI) can valuably contribute in addressing the first issue by providing essential features to analyze business management procedures and their broader implications on socio-technical systems, it falls short in addressing the second issue. Essential aspects of sustainability, such as the responsibility for potential future trade-offs through innovation, are not strategically integrated into the current framework. This study argues that without strategic integration of responsibility, there is a risk of contributing to a partially-sustainable—”irresponsible”—socio-technical system change as a result of business innovation activities. Therefore, an extended innovation process model for sustainability to embed responsibility at the core of innovation activities is required. For this purpose, the framework of Responsible Research and Innovation (RRI) is utilized. This paper reports on findings from a systematic literature review of a representative sample of empirical studies from the SI and RRI literature. Thereby, the goal was to extend the understanding of management opportunities within innovation processes for sustainability through the implementation of RRI principles, in order to create sustainable socio-technical systems.

## Introduction

Current developments, such as climate change, population growth and a widening economic gap between social groups continue to raise the significance of the sustainability agenda. Notably, this aspect has become increasingly relevant for innovation studies (Hallstedt et al., 2013; Berkhout, 2014) and is at the core of sustainability transition studies (Boons et al., 2013). The UN's Sustainable Development Goals (SDGs) have been formed in 2015 to put sustainability at the center of decision-making on the international scale. The SDGs suggest that action toward one goal will affect outcomes in relation to other goals, and it is stressed that development must balance social, economic and environmental sustainability. Thereby, the SDGs as a guide for innovation and research, illustrate not only the importance of clear goals to create sustainable socio-technical systems but also the high complexity across and between systems and their actors (Morton et al., 2017) which calls for a normative dimension for transitions toward sustainability (Schlaile et al., 2017).

Research that takes a multi-level perspective on sustainability transitions aims to analyze complex and long-term socio-technical transitions to sustainability and emphasizes the interrelationships between organizations and their broader environment (Geels, 2011). According to this line of thinking, organizations, including businesses, play a major role in the creation of socio-technical systems. Thereby, the way interconnected innovation processes are managed within businesses and the resulting outcomes have an impact on the overall system (Cantner and Pyka, 1998). However, our understanding of how organizations can manage innovation in a way that it contributes to creating environmentally, socially and economically sustainable socio-technical systems arguably remains limited.

The Sustainable Innovation (SI) literature (Boons et al., 2013; Berkhout, 2014) contains essential features that qualify the framework to analyze business management procedures and their wider implications on the socio-technical system (Boons et al., 2013; Hallstedt et al., 2013). Nonetheless, SI often fails to strategically integrate potential sustainability trade-offs, such as future conflicts between social and environmental sustainability aspects, into the innovation process and therefore has a non-holistic understanding of sustainability, in which not all aspects of sustainability are considered equally (Lubberink et al., 2017). Furthermore, the current literature on innovation processes for sustainability highlights the difficulty of including ethical considerations into innovation practices (Cuppen et al., 2019). Thereby, it remains open how to establish a more balanced view between technology development and social and ethical sustainability issues (Lubberink et al., 2017; Cuppen et al., 2019). Thus, despite the valuable analytical tools of SI, the framework needs to be extended to escape the risk of unintentionally contributing to partially-sustainable—irresponsible—socio-technical systems, which are e.g., more environmentally-friendly but socially unjust.

In order to broaden the current understanding of sustainability-related innovation, this paper suggests a responsibility extended innovation process for sustainability as a baseline to derive essential management implications. The framework of Responsible Research and Innovation (RRI) evolved as an emerging research field to address social needs and an orientation toward “right impacts” in research and innovation processes through the principles of inclusion, anticipation, reflexivity and responsiveness (Owen et al., 2013). Through its normative attributes, the RRI concept spurred a discussion about the purpose of research and innovation, underlying innovation goals as well as how these goals can be achieved ethically, inclusively, and democratically (Schomberg, 2012). Furthermore, RRI goes beyond current sustainability standards within systems and aims to integrate possible consequences into decision-making processes (Timmermans et al., 2017). RRI helps opening up alternative viewpoints that contrast with the standard linear thinking of innovation processes (Schlaile et al., 2018). Even though the underlying SI and RRI rationales promise to complement each other, an analysis of how the two frameworks can enrich each other in a joint innovation process model is currently missing (Lubberink et al., 2017; Cuppen et al., 2019).

To address this gap, this study aims to identify a set of management practices under which innovation processes for sustainability can contribute to socio-technical systems and complement them with insights from the RRI literature. This eventually contributes to responsible innovation process management for sustainability issues and thus impacts sustainable socio-technical system creation.

This study systematically reviews the empirical literature on SI and RRI regarding the management of innovation processes and derives a novel innovation process model, which embeds responsibility (as defined by RRI) in the innovation process for sustainability. Drawing from a dataset of ~360 peer-reviewed papers, this study subjects 29 empirical papers that meet all selection criteria and qualify for a final full-text analysis through inductive coding. Herewith, this study aims to answer the question: *how can innovation be managed to contribute to creating sustainable socio-technical systems?*

The following sub-tasks are considered to answer the research question. Based on the strategic considerations of Cooper (1988) to conduct a systematic literature review, important management practices within innovation processes for sustainability that are essential for the creation of socio-technical systems are identified in a first step. In a second step, it is analyzed how RRI principles can complement and extend management practices to achieve responsible management of innovation processes for sustainability. By using SI and RRI literature, the paper sheds light on (1) management practices, which appear to be relevant in order to understand how innovation processes for sustainability can influence socio-technical systems, (2) constraints within the important management practices for socio-technical system change in terms of responsibility, (3) insights from the RRI literature on how the innovation process for sustainability can become more responsible, and (4) insights on how managers can promote and organize innovation processes for sustainability in a responsible manner. Building on the sustainability-driven innovation process model from Keskin et al. (2013) the study concludes with a management proposal for a responsible innovation process model for sustainability. By implementing the findings from the literature review into an existing innovation process model for sustainability, it will extend the current knowledge in terms of responsibility aspects and provides perspectives for responsible management in the different innovation process phases. Ultimately, this can enhance management opportunities within innovation processes for sustainability through the implementation of RRI principles, in order to create sustainable socio-technical systems. Thereby, the management implications from the novel responsible innovation process for sustainability can more effectively address sustainability trade-offs, such as social and ethical sustainability dilemmas as well as future consequences of innovation activities. Furthermore, it can enhance the dynamic capabilities of innovation processes by indicating options for creating routines to transfer anticipated knowledge into current practices.

The study contributes to the literature in various ways. First, it extends current management opportunities within innovation processes for sustainability by adding RRI knowledge to contribute to the creation of sustainable socio-technical systems. Thereby, the understanding of sustainability is broadened in order to better account for all aspects of sustainability, such as social and ethical considerations, which are often difficult to capture in previous innovation process models for sustainability (Cuppen et al., 2019). Second, this study contributes to the RRI literature by improving its applicability to the business sector and extending the framework to go beyond the early stages of the innovation process (Lubberink et al., 2017). Thus, this study makes use of existing knowledge about the importance of business innovation processes for socio-technical system transition, the meaning of sustainable innovation to foster this transition and thereby aims to fill the research gap that important sustainability dimensions tend to be overlooked in current innovation process models for sustainability. The RRI concept is considered a high potential framework to address this task. In summary, insights from the SI literature regarding important management practices related to socio-technical system change will be identified and expanded by integrating RRI insights. This will eventually lead to a more responsible management approach of innovation processes for sustainability.

First of all, Section Literature background highlights the importance of the innovation process for socio-technical system change and current limitations in terms of responsibility, as well as ways RRI can address these limitations. In Section Methods, the systematic literature review method will be explained in more detail. The results presented in Section Results are structured according to the sub-tasks for answering the research question as outlined in the method section. The results suggest that responsibility can particularly enrich the innovation process for sustainability by introducing a strategic framework for external exchange and the integration of key stakeholders that often have been overlooked in the SI-driven approach, as well as an approach for addressing normative considerations that can better capture future developments and thus make innovations more robust to system dynamics. These mechanisms can, among others, assist in the creation of sustainable socio-technical systems. Based on the results, Sections Interpretation of the results in an innovation process for sustainability to derive concrete implications for responsible management and Conclusion and suggestions for further research provide a final assessment of the overall management implications from the responsible innovation process model for sustainability and discusses and concludes the paper by outlining a set of responsibility-related questions, which can guide decision-making during the different phases of the innovation process for sustainability.

## Literature background

This section aims to provide the background for deriving the relevant questions that guided the systematic literature review. Thus, it briefly discusses the important gaps in the relevant literature to which the present paper aims to contribute. Sustainability transformation research gained importance in times of wicked-problems (Turnheim et al., 2015; Schlaile et al., 2017). An important aspect of this literature is the consideration of the system as a complex arrangement of different actors at different levels (Geels, 2006, 2011). For SI as an emerging framework to address wicked-problems, this implies that “protagonists of the innovation will […] have to engage with the larger system in order to be successful, which may eventually lead to system innovations or transitions” (Boons et al., 2013, p. 2). Berkhout (2014, p. 290) furthermore argues that “sustainable innovation is linked to corporate strategy and that sustainable system innovation needs to be seen as a co-evolutionary process involving not only innovative firms but also a broader context of institutions, infrastructures, and consumer practices.”

However, despite the valuable contributions of the socio-technical system transition literature that puts sustainability at the center of attention (Geels, 2011), the literature to date provides more information about overall innovation systems dynamics and less about what this means for the management along innovation processes (Loorbach et al., 2009; Jacobsson and Bergek, 2011; Inigo et al., 2017; Ampe et al., 2021). Moreover, after an observation of the literature on “innovation processes for sustainability” and management implications, the tendency emerges that the sustainability management literature primarily focuses on optimizing innovation ecosystems to increase competitiveness and capability of innovation processes in a backward looking attempt (Mousavi and Bossink, 2017; Karlsson et al., 2018; Hansson et al., 2022) and less on options to improve the influence on sustainable socio-technical system change in a forward looking attempt. This, however, is essential to address social and ethical questions related to innovation (Cuppen et al., 2019; Sonck et al., 2020). Therefore, the systematic literature review aims to identify management practices along innovation processes for sustainability that are relevant to contribute to socio-technical systems. The SI literature is seen as a suitable starting point for this task. The concept aims to increase sustainability performance in products, processes and services (Boons et al., 2013). Thereby, it focuses on radical innovation to contribute to socio-technical system change (Charter et al., 2008). At this level, drastic transformations of current customs can take place (Hellström, 2007). The SI literature provides the setting to derive concrete management practices that can be seen as a foundation for influencing customs at socio-technical system level (Boons et al., 2013; Keskin et al., 2013; Berkhout, 2014). However, there are studies that question the assumption that SI sufficiently incorporates all aspects of sustainability, such as the responsibility about future consequences to ensure social sustainability (Hallstedt et al., 2013; Cuppen et al., 2019; Emilsson et al., 2020). This poses the risk that innovation processes for sustainability contribute to partially-sustainable socio-technical systems in which not all sustainability aspects are equally considered.

RRI, as another literature stream that focuses on innovation-led contributions to sustainable development, puts responsibility at the center of decision-making to address possible social and ethical issues in a forward-looking manner (Schomberg, 2012; Lubberink et al., 2017). Even though the term responsibility is often interpreted and defined differently in innovation studies, it generally describes responding to societal needs and striving for the “right impacts” (Owen et al., 2013, p. 28; Schomberg, 2012, p. 41). The main argument for this approach builds on the “dark side” of innovation. According to Coad et al. (2021, p. 103) this implies that “innovation is not always a force for good.”

Some authors already suggest important synergies between the SI and RRI frameworks (Lubberink et al., 2017; Cuppen et al., 2019). However, apart from this call, it has not yet been analyzed how the two frameworks can mutually enrich each other in a joint innovation process model. This will be explored with the following systematic literature review by examining how the RRI principles can be expressed in innovation processes for sustainability, particularly with regard to the important management practices for creating socio-technical systems. Building on insights from the empirical SI and RRI literature, it will become clearer how responsibility can evolve into a central component of innovation management along the entire innovation process, ultimately paving the way for social desirability to become a key factor in the creation of socio-technical systems.

## Methods

A systematic literature review on empirical SI and RRI papers was conducted in order to identify the key management practices within innovation processes for sustainability that are especially evident in the creation of socio-technical systems in order to subsequently improve them in terms of their responsibility attributes. This allows to deduct management implications for responsible management of innovation processes for sustainability to create sustainable socio-technical systems. The research procedure followed Cooper (1988) stages of conducting a literature review: Problem formulation, data collection, data evaluation, and analysis and interpretation.

The problem formulation started with the definition of a clear review goal. In this case the leading goal was to identify management practices within innovation processes for sustainability that can influence socio-technical system change and current concept failures that hinder responsible management of innovation processes for sustainability. The underlying assumption is that if, firstly, responsibility becomes embedded in innovation processes for sustainability and, secondly, innovation processes for sustainability can realize their abilities to influence socio-technical systems, then the creation of socio-technical systems will become generally more sustainable as the risk of overlooking important aspects of sustainability will be reduced. This formed the basis for the research question that guided the literature review: *How can innovation be managed to contribute to creating sustainable socio-technical systems?* Based on this research question the following sub-questions were formulated:

1. Which management practices are highlighted in the SI literature as effective for enabling innovation processes to contribute to socio-technical systems?
2. Regarding the management practices that are important to contribute to socio-technical systems, do innovation processes for sustainability incorporate responsibility and if not, why?
3. Based on the empirical RRI literature, how can RRI principles be expressed in innovation processes for sustainability, especially with respect to the important management practices to change socio-technical systems?
4. What information does the empirical SI and RRI literature provide to make responsibility a central part of innovation management?

Corresponding to the research questions a qualitative approach was chosen. As a second step of the problem formulation, the criteria for exclusion and inclusion were formulated. Only studies which met all search criteria were included for the further analysis (Table 1). An essential factor for the relevance of an article was its informative value about management implications along innovation processes. In addition, it was important that the innovation process had a clear link to sustainable innovation and originated from the RRI or SI literature (see criteria 5, 6, and 8). Moreover, it was necessary that the article used primary data e.g., data from workshops, interviews, etc. (see criteria 7). The data had to be unique and not overlap or coincide with other data from other papers (see criteria 3, 4). Finally, it was necessary that the article was published in English language and that the publication period was in a certain phase from 2013 to 2020 to ensure a clear search delimitation (see criteria 1, 2).

**Table 1:** Search criteria.

	**Inclusion criteria**
1	The report was written in English
2	The report was published between 2013 and 2020
3	The data of the report does not overlap with data from another report
4	If separate reports use the same data set, only the report with the most comprehensive reporting is included
5	The study reports on innovation processes or allows to draw conclusions about RRI or SI innovation processes and its management
6	The study is written from an RRI or SI perspective
7	The report used primary data (e.g., interviews, workshops, etc.)
8	The report focused on sustainable innovation

The objective of the systematic review was to combine information about two existing concepts, which also determined the setting of the search criteria and the applied search terms. Accordingly, the period of analysis begins with the year from which the concepts were used more frequently in the literature. Even though SI appears earlier than 2013, this year is considered the reference point for RRI (Stilgoe et al., 2013). In order to draw a clear line of analysis, end of 2020 was chosen as the final point of analysis. In addition, the search terms only included specific keywords that describe the concepts and related keywords were omitted in order to prevent a dilution of the results.

**Figure 1:**
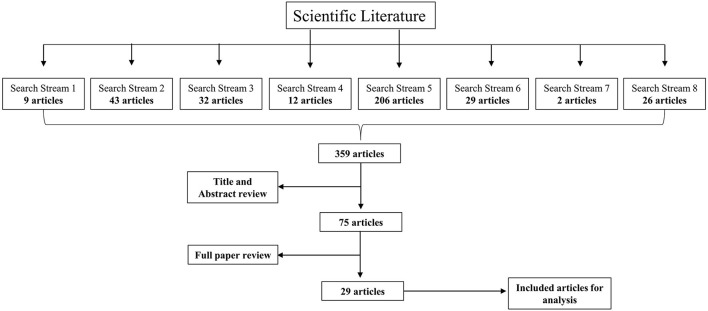
Research procedure. Using the predefined search terms, the systematic literature review began with a total of 359 articles. The boxes in the first row indicate which search term produced how many results. Two screening rounds followed: First, titles and abstracts were screened, and articles that did not meet the inclusion criteria were excluded. Second, the full articles were screened. Finally, 29 articles met all search criteria (see Table 1).

The research was conducted in June 2021 using Web of Science as electronic database. For data generation the following search terms were employed in “all fields”: “Sustainable Innovation” AND “Responsible Research and Innovation” (1); “Sustainable Innovation” AND “Innovation process^*^” (2); “Responsible Research and Innovation” AND “Innovation process^*^” (3); “Product innovation process” AND “Sustain^*^” (4); “Innovation process” AND “Sustainability” OR “Sustainable” (5). Most of the studies concerned with the topic of sustainable innovation and responsibility occurred in the areas of green sustainable science, environmental science as well as management. In addition, a journal-specific analysis was performed in order to strengthen the data base. The journals “Sustainability” (6), “Journal of Cleaner Production” (7) and “Journal of Responsible Innovation” (8) were selected because more than 50% of the publications that used the search terms were published in these journals. Therefore, this was considered a suitable basis to further strengthen the data base in a purposive manner by selecting a representative set of articles. Figure 1 outlines the research procedure.

In total, 359 papers corresponded to the search criteria. After a first screening round of the study abstracts, 75 papers remained. Most of the papers were disqualified due to a lack of empirical research and the absence of primary data. Additionally, three articles were included because of their thematic relevance to answer the research question. In the second screening round, the full texts of the 75 papers were reviewed, resulting in a total of 29 papers that qualified themselves for further analysis. The main reason for exclusion in the last screening round was a poor focus on sustainable outcomes and a lack of tangible insights about the innovation process.

The thematic analysis of the papers was performed with the help of the Citavi, MAXQDA, and Excel. The research questions served as a guide for the analysis—specifically, inductive coding was used to analyze each sub-question in order to answer the overall research question:

Sub-question 1: What are the management practices within the innovation process for sustainability that are important for socio-technical system change?
Emerging sub-categories:
  - Reconciliation of economic profit and sustainability
  - Strategic leadership
  - Systemic perspective
Sub-question 2: Regarding the management practices that are important to contribute to socio-technical systems, do innovation processes for sustainability incorporate responsibility and if not, why?
Emerging sub-categories:
  - Dominance of economic profit
  - Complexity issues
  - Missing inclusion

In a second step, the insights from the RRI literature were mapped to the categories that have emerged. This involved examining how the RRI principles were used to support the innovation process in the analyzed empirical RRI papers and finally how this influences the general innovation process for sustainability in terms of the established categories.

To derive management implications from a responsible innovation process for sustainability, essential relationships between the RRI and the SI concepts were established. The goal was to extend the understanding of opportunities within innovation processes for sustainability to implement RRI principles in management practices, in order to create sustainable socio-technical systems. This was done by identifying meaningful statements. Empirical claims about the effects of RRI principles on the innovation process were highlighted word-for-word in a spreadsheet and in the next step put into the thematic categories in order to interpret them as groups (Table 2). The final synthesis was further influenced by the innovation process model for sustainability as outlined by Keskin et al. (2013). In the eyes of the authors, this is currently the most developed innovation process for sustainability that also fulfills the conditions for achieving the research objectives of this paper. Thus, the model, which is based on radical innovations, can be used to derive concrete management implications that are intended to contribute to socio-technical system change. However, the model suggests that aspects of responsibility are not part of the analysis, which leaves room for extension. Nevertheless, the model helps to further interpret the results to provide advice for responsible management of innovation processes. The insights gained from the systematic literature review will be further explored in the following section.

**Table 2:** Illustrative alignment of framework through inductive coding.

**Empirical claims from the RRI literature**	**RRI mechanisms**	**Areas of influence within the innovation process for sustainability**
“The findings confirm that arguments on impacts brought up by the participants serve for considering implications in dissemination and use of new products in the prospective reflection of the innovation path […]. The identified socio-economic aspects raised by the citizens prove that risks of the technology are to a large extent systemic risks, which can only be regarded in their social interplay with society […]” (Ketzer et al., 2020, p. 206).	– Inclusion – Anticipation	Sub-question 1: – Strategic leadership – Systemic perspective
“[…] we have observed that only firms with clear moral motives will integrate these label criteria in their innovation policies. These innovation policies will then support the uptake of moral objectives during the product development process […]. Firms with primarily instrumental motives will see the label criteria as a translation of the consumer demand for ‘health' and will only use them if this consumer demand provides increased sales” (Garst et al., 2017, p. 18).	– Reflexivity – Responsiveness	Sub-question 1: – Strategic leadership Sub-question 2: – Dominance of economic profit
“The responsive and adaptive character of the SMEs' innovation processes becomes evident in their ability to quickly adapt to changing market conditions. The high degree of flexibility was indeed considered essential for succeeding in the market and meeting their customers' interests; an ability that often distinguishes them from their larger competitors” (Auer and Jarmai, 2018, p. 7).	– Responsiveness	Sub-question 1: – Reconciliation of economic profit and sustainability

## Results

### Insights into managing innovation for sustainability from the SI literature

#### Important management practices in innovation processes for sustainability to contribute to socio-technical system change

The SI literature offers valuable insights into important management practices that enable innovation processes to influence socio-technical systems. Thereby, three main aspects stand out. They will be further explained in the following and are summarized in Table 3.

**Table 3:** Management principles within innovation process for sustainability that contribute to socio-technical system change.

**Management principles of the innovation process for sustainability**	**Described mechanisms (tools)**	Type 1^*^: Outcome in terms of socio-technical system creation
Reconciliation of economic profit and sustainability	• Introduction of sustainability metrics that influence search heuristics and support strategic decision-making. • Creation of competitive business cases that combine sustainability with profit.	• Increased profitability through sustainability and thus sustainability as a competitive factor. • Increased directionality that influences the interaction with partners.
Strategic leadership	• Clear sustainability goals defined at the beginning of the innovation process. • Consideration of innovations under a long-term perspective. • Creation of a high-quality network for knowledge generation. • Ongoing testing and evaluation of innovations in target groups to capture needs. • Willing and committed management that influences the overall value system of the company.	• Early response to potential systemic movements ensures long-term economic viability and acceptance. • Strategic sustainability plan as orientation for decision-making and thus efficiency throughout complex and dynamic innovation processes. • Sustainability as an integral part of the business image creates a common corporate drive and guides communication with external partners.
Systemic perspective	• Consideration of innovations under a systemic lens. • Diverse alliance of partners (internally and externally). • Creating innovation demand along the value chain.	• Creating infrastructure preparedness. • Increased ability for complex decision-making. • Developing interconnected innovations along the whole value chain.

* Type 1 indicates that no integration of RRI principles has taken place yet.

The first category that emerged is the reconciliation of economic profit and sustainability. SI represents an option for businesses to link profit with the desire to contribute to sustainability (Ligardo-Herrera et al., 2018; Emilsson et al., 2020). Kennedy et al. (2017) underline this by focusing on the effects of radical sustainable innovation on the innovation process and thereby highlight sustainability metrics as an important factor. In this case, sustainability is placed alongside low cost and high product quality in order to optimize product outcomes and gain competitive advantage. As soon as sustainability dimensions influence search heuristics, they become one of the main strategic management elements, which direct decision-making. This is further highlighted by Björklund and Forslund (2018). In all six case studies among Swedish retailers and logistics service providers, the creation of strategic sustainable business cases, that ensure a win-win situation between sustainability and competitiveness was highlighted as one of the main reasons for success. This enables the businesses to contribute to a sustainable transition without negatively impacting profit opportunities. In summary, a “proactive sustainability strategy” can harness the engagement with external actors and influences the way businesses deal with their infrastructure in a sustainable way. This in turn, increases the impact on creating socio-technical systems (Kennedy et al., 2017, p. 721).

A strategic leadership that assures sustainability throughout the entire innovation process is the second management aspect. Herewith, SI emphasizes stewardship and strategic management. On the one hand, stewardship can be established by defining clear sustainability goals. Keskin et al. (2013) describe goals as intended values, which are formulated at the early stages of the innovation process. These can create guidelines for decision-making and can influence the entire organization at different levels, until the organization establishes a shared organizational drive. Management, on the other hand, involves various aspects: First, management includes the application of strategic tools, such as life cycle analysis (LCA) or backcasting, which assists in the evaluation of innovations in terms of future developments and increases efficiency in processes (Hallstedt et al., 2013; Keskin et al., 2013). Second, management describes clear procedural steps that are placed at the different phases of the innovation process. An important task is to formulate a sustainable business case at the beginning of the innovation process (Björklund and Forslund, 2018). In this context, various aspects are considered in order to make a sustainable and successful business case. First, innovations are considered under a long-term perspective to enable an early response to possible movements in order to create long-term economic value. Second, Emilsson et al. (2020) emphasize the importance of networking in order to acquire external knowledge and thus increase the chances of success. Thereby, competition is seen as an important driver for creating high-quality networks, finding potential clients, as well as acquiring financial resources. Competition, in turn, can increase credibility and thus increase acceptance, knowledge acquisition, and the position in debating business cases (Keskin et al., 2013; Kennedy et al., 2017; Emilsson et al., 2020). The integration of external resources can serve as an additional success factor. By testing and evaluating innovations, it is possible to determine whether current business ideas are accepted by end users or not. This provides an opportunity to capture needs and the societies' willingness to purchase final outcomes (Hallstedt et al., 2013). Third, SI highlights the “willingness” of the management to commit to sustainability. This is often understood as a shared mission or vision that forms the basis for decision-making and directs innovation processes in a goal-oriented manner (Hallstedt et al., 2013). The commitment of the management is relevant in order to provide a strategic sustainability plan. In this case, the management is not only responsible to communicate the vision internally, but also toward external stakeholders. This requires a management that is willing to accept only those results as “right outcomes” that are consistent with the defined goals (Hallstedt et al., 2013; Keskin et al., 2020). According to this, predefined goals help to guide management practices toward socio-technical change. This process can be initiated when goal-oriented management leads to the formation of strategic cooperation and co-development. Herewith, allies help to address complex innovation challenges at a systemic level (Hallstedt et al., 2013; Lubberink et al., 2017).

The aim of achieving “right outcomes” is leading to the third aspect: the assessment of technological products as a part of a wider socio-technical system. Hallstedt et al. (2013) define this as the “broader societal systems being prepared for these products […] [as] products rely on infrastructure that is outside of […] [the producers] direct control” (Hallstedt et al., 2013, p. 282). Infrastructure can describe many things, from financial infrastructures to education (Panciroli et al., 2020). In the SI literature, the majority of innovations that are referred to as “right outcomes” are those that are able to address complex challenges, as well as products that are developed through a systemic lens (Keskin et al., 2020). In order to address complexity issues, SI highlights a diversity approach, in which a complex alliance of actors, such as employees, specialists and external consumer cooperate with each other (Björklund and Forslund, 2018). Keskin et al. (2013) demonstrate, that firms which operate in mono-disciplinary teams have more difficulties in managing system complexities. This could be due to the fact that network formation in these companies tend to rely more on *ad-hoc* decisions and less openness to new contacts, compared to multidisciplinary teams. Here, teams grow with their innovations and solve many sub-tasks in-house. With this finding Keskin et al. (2013) support the assumption that strategic networks are important for socio-technical system change, it is however added, that this highly depends on the internal knowledge about the individual innovation. Kennedy et al. (2017) further point out, that the innovation process should aim to create demand along the entire value chain in order to push socio-technical system change. Thus, innovations should not be considered independently from their environment but should consider different and co-existing needs. Khan et al. (2016) draw on this by looking at the financial infrastructures of sustainable innovations and conclude, that the mindset of the financiers must also change in order to enable a sustainable transition with the help business innovation.

#### Challenges in managing innovation processes for sustainability

The SI literature offers insights into existing management challenges that prevents it from becoming responsible management. Most importantly, this negatively impacts the identified management principles that are important for contributing to socio-technical system change, and therefore risks contributing irresponsibly to the creation of socio-technical systems. A summary of the main challenges is shown in Table 4.

**Table 4:** Shortcomings of SI in terms of responsibility.

**Main barriers for responsible management of innovation processes for sustainability**	Type 1^*^: Outcome
Dominance of economic profit	Dominance of the economic model. In times of critical decision-making (e.g., crisis) economic aspects are prioritized over other sustainability aspects, such as social and ethical aspects. Focus on technological solutions over efforts to address reasons that might cause a certain problem.
Complexity issues	Increased management complexities that impedes efficient internal and external communication practices. External insights fail to be re-integrated into the innovation process.
Missing inclusion	Too late or incomplete integration, resulting in one-sided representation of stakeholders. Limiting the system perspective. This can lead to out-of-context innovations that are not required or desired by the affected society.

* Type 1 indicates that no integration of RRI principles has taken place yet.

The SI literature often points out that the strong influence of profit and competitiveness on innovation processes dilute sustainability. Emilsson et al. (2020) describe that the focus on the economic model leads to an underrepresentation of social perspectives. Khan et al. (2016) point out an additional issue. Innovation processes often concentrate on technological aspects and how they can be further improved to answer specific market needs. Thereby, the more time-consuming investigation of the reasons that might cause market needs moves into the background. This is further supported by findings from Keskin et al. (2013) who point out that in times of critical decision-making, sustainability-minded companies tend to choose the more cost-effective option and thus act at the expense of their initial sustainable goal. Among other things, this leads to a mismatch between the intended and the created value.

One reason for this phenomenon could be linked to increased process costs that arise when a company chooses the more complex but sustainable path. In the presence of efficiency and economic profit, this path loses attraction. In terms of complexity, Björklund and Forslund (2018) point out another challenge. When processes become more complex, it becomes more difficult for the management to balance the volume of information inflows without compromising the competitiveness of innovations. Therefore, user requirements and market insights are often bypassed, as this can increase the complexity of the innovation process (Keskin et al., 2013). One solution to manage innovation processes with high complexity would be efficient internal and external communication. However, the reviewed studies indicate, that communication between stakeholders, such as product developers and suppliers, but also within businesses, often remain poor (Emilsson et al., 2020; Pakura, 2020). Thus, important information is not fully communicated and thereby can create potential mistrust and less sensitivity for existing market needs (Hallstedt et al., 2013; Björklund and Forslund, 2018). In addition, insufficient communication risks overlooking future consequences of innovation. This can be most effectively addressed by improved knowledge management and strong engagement with different stakeholders (Emilsson et al., 2020).

The inclusion of external stakeholders at the early stage of the innovation process could favor engagement. However, the insights from the literature review only indicate a limited engagement of sustainability-minded businesses with external actors (Hallstedt et al., 2013; Emilsson et al., 2020). Even though the importance of stakeholder integration is widely confirmed by the papers, clear gaps emerge (Hallstedt et al., 2013; Auer and Jarmai, 2018). For instance, a late integration leads to reactive innovations or a very limited and uniform integration (Hallstedt et al., 2013; Keskin et al., 2013; Pakura, 2020). This can lead to context-insensitive innovations that are implemented without capturing the market sentiment (Keskin et al., 2013; Emilsson et al., 2020). In other words, without an engagement strategy for sufficient inclusion, a clear framework for socially desirable decision-making and opportunities to create routines for knowledge management along the innovation process, innovations will run the risk of compromising responsibility.

One possible approach that can provide strategic support is RRI. In the next chapter, it is examined how the RRI principles can affect the innovation process. The analysis is divided into the four principles of RRI: inclusion, anticipation, reflexivity and responsiveness.

### Insights for responsible management of innovation processes from the RRI literature

To enable innovation processes for sustainability to increase their impact on socio-technical systems to contribute to the creation of sustainable socio-technical systems, insights from the RRI literature can be used. Schomberg (2012, p. 50) describes RRI as “a transparent, interactive process by which societal actors and innovators become mutually responsive to each other with a view to the (ethical) acceptability, sustainability and societal desirability of the innovation process and its marketable products.” The following section provides results from the reviewed empirical RRI papers and how the RRI principles influenced the entire innovation process.

#### Inclusion

A strategic inclusion is often seen as a way to reduce uncertainty in business innovations. This is especially important at the beginning of the innovation process in order to acquire relevant knowledge about the context and market needs. Ketzer et al. (2020) reveal that the inclusion of local participants as local experts can help to turn potential conflicting goals into valuable compromises. This can increase the general acceptance. Acceptance, in turn, is an important success factor for innovation. Panciroli et al. (2020) emphasize the need for inclusion in order to act ethically and achieve acceptance. Thereby, ethical aspects can help to set standards for decision-making, which can lead to high-quality products. Thus, asking ethical questions early on in the innovation process but also during later stages can have positive effects on business success. Chatfield et al. (2017a) further strengthen this argument. Here, an advantage is seen in addressing risks resulting from innovation at an early stage and incorporating these findings later on in the innovation process. This can increase flexibility and reduce costs by uncovering problems early, which otherwise would have occurred at a later and more cost intensive stage. However, there is a chance that not all risks will be captured. To address this concern, the importance of diverse decision-making processes is emphasized. The quality of risk assessment increases with the involvement of a broad and diverse group of decision-makers, that allows different perspectives on one topic (Jakobsen et al., 2019; Panciroli et al., 2020).

#### Anticipation

In general, anticipation leads to greater awareness of potential future impacts (Brier et al., 2020). Long et al. (2020) describe anticipation as having a systems perspective that helps to identify various social and ethical sustainability issues early on. Thereby, anticipation can influence the strategy of the entire innovation process. With anticipation potentially changing circumstances can be identified in a dynamic procedure. This increases the likelihood that the product will remain competitive in the future. Besides the advantage of increasing consumers' future approval for products, Timmermans et al. (2017) also highlight the possibility of shaping future standards through anticipation. In this case, future market standards or regulations are evaluated before they are applied. This gives companies the chance to better prepare in advance. Companies also have the chance to co-determine future regulations or go beyond current regulations in order to differentiate themselves from competitors.

#### Reflexivity

Reflexivity is important to enable critical thinking about underlying values and motivations as well as to reflect on existing knowledge and compare this knowledge with perceived realities (Lubberink et al., 2017). After reflection, the gained knowledge should impact the management of the innovation process and stimulate possible modifications (Brier et al., 2020). An open and transparent innovation process is seen as a key mechanism to continuously provide options for reflection and association (Lubberink et al., 2017). Ketzer et al. (2020) suggest that reflection alone is not sufficient if the public does not have the opportunity to re-evaluate the previously reflected results. Reflexivity is therefore not a single event but a repetitive task. Again, the dimension of inclusion becomes important. The involvement of external stakeholders can challenge existing mindsets and thereby contribute toward a rethinking of practices. A significant advantage of this repetitive activity is the ability to create routines, which in turn can improve adaptability. Thereby, routines influence the overall effectiveness of the innovation process and thus can minimize process costs (Gurzawska et al., 2017).

#### Responsiveness

Responsiveness goes beyond deliberation, which is when innovators are willing and able to take responsibility in order to address social or ethical issues (Lubberink et al., 2017). With responsiveness, innovators and other system actors become responsive to each other (Lubberink et al., 2017; Ligardo-Herrera et al., 2018). “Transformative mutual learning” (Brier et al., 2020, p. 563) strengthen the understanding of different expectations but also requires a high degree of responsiveness. This illustrates that responsiveness means, on the one hand, the ability to respond to public perspectives and, on the other hand, to implement them. Auer and Jarmai (2018) consider responsiveness as an advantage as it favors a quick adaption to changing conditions, such as changing market requirements. Here, it is further emphasized that the resulting flexibility is essential for smaller companies to generate competitive advantage.

The analysis of the impacts of inclusion, anticipation, reflexivity and responsiveness on innovation processes reveals that the principles cannot be considered separately. Instead, they need to be seen as interrelated and mutually influencing. Furthermore, the examples show that responsibility can become an integral part of the innovation process and thereby influence final outcomes. The reviewed empirical RRI papers suggest that the principles can be used as an instrument to gain competitive advantage.

### Synthesizing insights from the RRI literature and their implications for innovation processes for sustainability

After the previous sections have outlined (1) management practices within the innovation process for sustainability, which are important to create socio-technical systems, (2) limitations in terms of integrating responsible management practices, as well as (3) insights from the RRI literature into how the principles can complement the innovation process for sustainability regarding these two aspects, the following table combines these three parts (see Table 5). The information on the left-hand reflects the insights from the RRI papers and how the principles affected the innovation process. The information on the right-hand shows the key features of the inductive categories for each sub-question, and how they are influenced when the RRI principles are applied. Thereby, Table 5 helps to better understand the relationships between the individual themes. It furthermore illustrates, how the RRI principles can support the innovation process for sustainability to create sustainable socio-technical systems. For instance, the category of reconciliation of economic profit and sustainability as a management practice that has an influence on socio-technical system creation, can be further supported by the RRI principles inclusion and anticipation. These two principles help to go beyond current standards (e.g., sustainability standards) and thereby extend the possibility to influence future regulatory developments, which in turn can favor current innovation directions.

**Table 5:** Contribution of RRI principles on innovation processes for sustainability.

**RRI**					**Innovation process for sustainability**
**Active principles** ^*^				**Impact on the innovation process initiated by the principles**	Type 2^**^: Implications for responsible management of innovation processes for sustainability
**Inc**.	**Ant**.	**Ref**.	**Res**.		
√	√			Going beyond current (sustainability) standards and thereby increasing competitive advantage.	Involvement in future regulatory developments that can benefit the company's own success.
√	√	√	√	Responsibility as a moral code of conduct directing decision-making.	Long-term directionality for strategic leadership during transformation processes.
√	√			Evolving from public acceptance to public desirability	Reduction of uncertainties regarding future developments and thus better alignment with market needs.
√	√	√	√	Responsibility as a design element that influences the entire innovation process and the final outcomes.	Additional options to differentiate themselves from competitors.
		√	√	Contribution toward building up a shared company value system and strengthened communication.	Efficient decision-making about complex issues (e.g., sustainability dilemmas) supported at different levels of the company.
√	√			Mapping of a holistic system and thereby identifying potential gaps.	Creating system and infrastructure preparedness from a multi-stakeholder viewpoint.
√	√	√	√	Enhancing adaptability toward changing market conditions.	Ability to efficiently deal with socio-technical system dynamics.
√	√			Forming a research centered strategic alliance of partners.	Developing interconnected innovation which integrate various actors along the value chain to push systemic changes.
√	√	√	√	Responsibility as a moral conduct that provides guidelines in times of critical decision-making and limits the risk to overprioritizes economic aspects.	Counterbalance to the dominance of economic agendas and the bias toward profit in phases of critical decision-making.
		√	√	Establishing routines for sufficient communication and information transfer.	Complexity issues that can disrupt communication can be addressed through increased adaptive capacities.
√	√			Guidelines that favor a strategic inclusion and engagement plan.	Assistance through a strategic engagement framework that favors fruitful inclusion.

* RRI principles inclusion, anticipation, reflexivity, and responsiveness.

** Type 2 indicates that an integration of RRI principles into the existing categories has taken place: Sub-question 1 = reconciliation of economic profit and sustainability; strategic leadership; systemic perspective. Sub-question 2 = dominance of economic profit; complexity issues; missing inclusion.

The information provided in Table 5 demonstrates that responsibility as defined by RRI can contribute to the innovation process for sustainability. Furthermore, the table indicates that the integration of responsibility dimensions in innovations processes for sustainability does not necessarily make the process more complicated. This is supported by some of the reviewed empirical SI paper, such as those connected to health innovations, which highlight that certain responsibility aspects are already unknowingly part of the sustainability-oriented innovations (Emilsson et al., 2020). Instead, the integration of RRI principles can even be beneficial. They, can support the innovation process for sustainability by strengthening already existing management practices that contribute to the concepts importance for socio-technical system creation (Timmermans et al., 2017).

When responsible management of innovation processes is approached from an SI perspective, it requires accompanying the entire innovation process, including commercialization, to support the creation of sustainable socio-technical systems. The reviewed papers provide insight into that matter. Most of the analyzed articles described responsibility as a moral umbrella which can influence decision-making (Chatfield et al., 2017b; Garst et al., 2017). Garst et al. (2017) suggest to define criteria of responsibility as “criteria […] could help in translating the moral motives to design requirements, acting as a tool for defining […] product composition” (Garst et al., 2017, p. 18). Through a strategic and gradual integration of moral design requirements into process phases, concrete responsibility standards can emerge. These responsibility standards can be translated into goals that guide the entire innovation process for sustainability. Timmermans et al. (2017) argue similar to Garst et al. (2017) and highlight responsibility measures in form of requirements (gates) that need to be reached after every process stage in order to reach the next stage. This would allow responsibility standards to become an outcome or design element. Sonck et al. (2020) highlight meta-responsibility to keep the innovation process on track and to achieve positive societal impact. In this study, normative and procedural tools are elaborated. Normative anchor points are used to align project goals with the sustainability guidelines of the corporate strategy. The corporate strategy in turn focuses on major global challenges. The procedural tools are linked to the normative tools. The procedural tools use the normative goals to guide stakeholder integration. They accompany the engagement process and are presented to the stakeholders in order to obtain targeted feedback. This steers the innovation process in a responsible direction and enables targeted input.

The reviewed RRI papers do not provide specific information how socio-technical systems are created with the help of RRI related management in innovation processes. The analysis of the RRI papers often focuses on incremental innovation rather than radical innovation (Cuppen et al., 2019; Jakobsen et al., 2019). Therefore, the infrastructure and the overall socio-technical system does not play a major role. In contrast to that, “standardized principles about public governance of research and innovation” (Jakobsen et al., 2019, p. 2) are more prominent. It appears, however, that RRI principles, such as anticipation, allow to go beyond current assessments of socio-technical systems and enable to include future developments into current innovation process strategies (Brier et al., 2020). This can create new possibilities in terms of developing interconnected innovation and system preparedness. In a best-case scenario, this would lead to the creation of a strategic network, to the co-determination of future settings, such as regularities, and ultimately to the provision of long-term oriented solutions. The study from Timmermans et al. (2017) illustrate how such a scenario could look like. In this study the authors describe a case in which gouging “the mood of the public and [by going] beyond market standards […] responsibilities emerged that implied attending to the needs of elderly people and safeguarding the privacy of users by deploying “Privacy by design” (Timmermans et al., 2017, p. 325).” This in turn influenced how the “companies engaged with external actors to establish requirements and norms to their R&I processes” (Timmermans et al., 2017, p. 325) and demonstrates how responsibility can become a strategic design element and thereby a tool to create sustainable socio-technical systems through anticipation in innovation processes for sustainability.

In general, it becomes evident that the SI and the RRI frameworks have different approaches which could set the foundation for socio-technical system change. While SI takes a more goal-oriented approach (Hallstedt et al., 2013; Keskin et al., 2013), RRI is based on an inclusive-oriented approach with the society (Owen and Pansera, 2019). However, it especially appears that the fusion of the two frameworks can yield benefits in terms of sustainable socio-technical system creation. Insights from the RRI literature can enrich innovation processes for sustainability by introducing a strategic framework for external exchange and for the integration of key stakeholders, that might be overlooked in a purely SI driven approach (Lubberink et al., 2017). Furthermore, anticipation can help to uncover existing system interrelationships and better anticipate future developments and consequences (Timmermans et al., 2017). This increases the likelihood of finding long-term ethical solutions, identifying conflicting goals and finding suitable solutions at an early stage. These mechanisms can, among others, assist in the creation of sustainable socio-technical systems. In terms of management, the RRI principles can contribute to SI in overcoming some of the currently existing management challenges by providing further tools for decision-making especially in times of critical decision-making (e.g., in times of crisis). This can minimize the risk of losing sight of market needs in the run of the innovation process (Keskin et al., 2013). Furthermore, RRI can help companies to better align their intended values with their created values during the process. Finally, a strategic integration of the principles reflexivity and responsiveness can establish communication routines, which in turn can influences the overall effectiveness along the innovation process for sustainability, which in turn can influence innovation success (Gurzawska et al., 2017; Auer and Jarmai, 2018).

Certainly, the integration of responsibility aspects into innovation processes for sustainability is not risk-free. In this respect, it must be mentioned that the integration of responsibility can increase the complexity of the innovation process and thereby can cause lower effectiveness and higher process costs. The integration of a very broad public can lead to problem-unspecific information which makes it harder to manage incoming knowledge (Pakura, 2020). However, it can be counter-argued that responsible management can also increase prospects of public acceptance and thereby reduce the risk of failure, which in turn can offset the increased process costs due to higher a complexity (Hallstedt et al., 2013). In summary, there is still a debate about whether the advantages or disadvantages of integrating RRI principles into business innovation prevail. The findings of this analysis, however, indicate that the impact of responsibility on innovation processes for sustainability can in fact be positive.

The following section aims to assess responsibility in innovation processes for sustainability in order to allow future research to deepen the understanding about the effects of responsible management on innovation processes of sustainability and socio-technical system creation. The model assessment builds on the innovations process model presented by Keskin et al. (2013) and is enriched with the findings obtained in the course of the systematic literature review. In the eyes of the author, Keskin et al. (2013) currently provide the most advanced innovation process model for sustainability.

## Interpretation of the results in an innovation process for sustainability to derive concrete implications for responsible management

Keskin et al. (2013) elaborate on the innovation process of new ventures driven by sustainability and how this goal affects organizations. It is assumed, that young entrepreneurs are important actors for implementing radical innovations and thus essential for socio-technical system change. The focus of the model on organizations that seek system change by going beyond established landscapes is one of the main reasons for choosing this model. The main difference compared to other well-known innovation process models, such as the stage gate model by Cooper (1990) is the “sustainability intention” as initial condition and the “value created” as functional endpoint (Keskin et al., 2013, p. 55). These two points reflect the need for the company to define clear sustainability goals at the beginning of the process and to verify if the final results meet the conditions of the original goals at the end of the process. Within the innovation process, external factors as well as internal factors influence the different stages.

**Figure 2:**
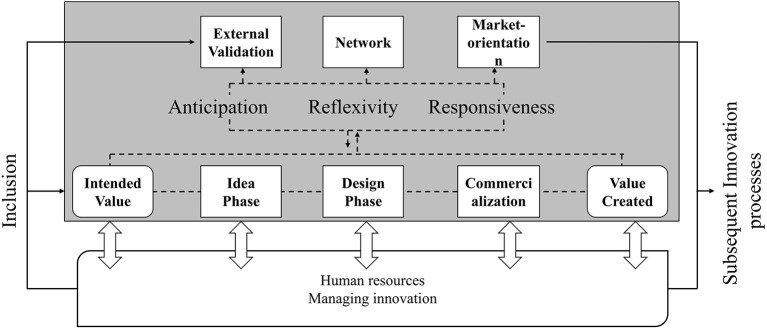
Assessment of responsible management in the innovation process for sustainability own assessment adapted from Keskin et al. (2013, p. 55). Based on the innovation process of Keskin et al. (2013). External validation, network and market-orientation are essential components that are influenced by responsible management of the innovation process. They also influence the management as a source of knowledge based on which the innovation process responds. The dashed line reflects the normative character of RRI. The RRI principles accompanies the entire process. Inclusion has a direct influence on further activities in the innovation process. The extent and variety of inclusion determine the results that emerge from anticipation and thus what is ultimately reflected and responded to. The transfer of knowledge gained to subsequent processes is determined by responsiveness, which requires that the experiences from previous processes are transferred to subsequent processes. The continuous line represents the continuation of experience in subsequent processes as part of the current innovation process.

According to Keskin et al. (2013) internal attributes (e.g., management and human resources) influence the innovation process for sustainability only in the design phase. Even though the management and the human resources are generally referred to as important, these two factors are only specifically highlighted in this phase. The reviewed RRI papers in this study highlight the importance of the management at every stage and especially in the idea and design phases (Ketzer et al., 2020). The management is, for instance, responsible for including a broad public perspective to the design of business cases. Inclusion, in turn, requires the know-how and willingness of the management to do so. Therefore, internal factors, such as management and human resources, are considered a main attribute at all levels of the innovation process. In the following, the responsible management framework for an innovation process for sustainability will be outlined. The listing is structured according to the pre-defined internal and external factors and enriched by the insights from the literature review on the management practices. The overall model is presented in Figure 2.

### Management and human resources

Ethical aspects are an essential part of the training portfolio within companies (Chatfield et al., 2017a; Brier et al., 2020). This internal sensitivity ensures that technological developments are always considered in correlation with their possible impact. For this purpose, future responsibility experts are recruited (Rivard and Lehoux, 2020). Herewith, a close cooperation with external stakeholders from other areas is required, such as university hubs which offer related responsibility programs (Pakura, 2020). Thereby, the company is establishing an internal value system by bringing employees who are sensitized to responsible issues into the company. Furthermore, the management acts as a responsible coordinator and provides responsible guidelines for the idea, design and commercialization phases (Hallstedt et al., 2013). The management is also prepared to assess responsibility standards at the individual phases of the innovation process.

### External validation

External knowledge generation in the idea phase relies on a diverse group of experts (Keskin et al., 2013). Local contexts are strategically deployed in the generation of innovation ideas. Thereby, affected individuals become local experts (Karlsson et al., 2018). Furthermore, multi-disciplinary teams both inside the team as well as outside the team are considered important (Björklund and Forslund, 2018). A particular advantage of this approach is the inclusion of actors that represent the broader socio-technical system. This allows to investigate the development of the innovation in the idea and design phase from different angles (Hallstedt et al., 2013) leading to a better identification of possible alternative strategies that may be more in line with the intended goals. To further strengthen the idea-phase, business cases that respond to social and environmental needs are tested in a competitive surrounding in order to gain fruitful feedback, generate interest, and engage with other professionals and potential competitors (Keskin et al., 2013; Jakobsen et al., 2019).

### Network

The creation of a high-quality network is favored by a management board that encourages competition, seeks external knowledge and feedback, and gets involved in research activities at an early stage (Hallstedt et al., 2013; Kennedy et al., 2017). Acquiring specific expertise does not only help to improve innovation, but also increases status and visibility in the network. This prompts other market participants to reflect on the observed behavior that surrounds them and whether individual benefits can be acquired. This, in turn, can propel overall socio-technical system change using the combined forces of a wide range of innovations and system actors. A strong position within the network helps to realize change processes (Kennedy et al., 2017). In addition, intense exchange with stakeholders helps to identify innovation opportunities along the value chain. This can be further supported by anticipatory and normative tools that visualize strategic advantages despite socio-technical system dynamics (Timmermans et al., 2017).

### Market-orientation

Especially in times where critical decision-making is required the management applies responsibility standards as a moral code of conduct (Garst et al., 2017). This provides an additional orientation parameter for decisions, such as on materials, integration strategies or possible supply chains, which leads to greater efficiency in decision-making processes without compromising sustainability aspects. In addition, market orientation is promoted. Through principles such as responsiveness in the design and commercialization phases, as well as reflexivity and inclusion along the entire innovation process, market-oriented perspectives are stabilized (Lubberink et al., 2017; Emilsson et al., 2020). Furthermore, the continuous re-integration of insights can create routines in dealing with market dynamics. Public engagement is combined with the provision of clear targets, which ensures the generation of valuable insights that relate to corporate targets (Gurzawska et al., 2017).

### Combining intended and created value

The management considers the innovation process for sustainability as a living process. Therefore, the management does not only compare the created value with the intended value at the end of the process in order to learn from the results, but also in order to re-integrate experiences into follow-up innovation processes. Process protocols, for instance, can help to capture decision-making processes, which simplifies the re-integration of knowledge (Gurzawska et al., 2017; Auer and Jarmai, 2018). This favors the overall learning curve as well as enables an institutionalization of routines. Routines, such as for responsive action, can help overcome linear thinking patterns during innovation processes and thus increase dynamic capabilities.

## Conclusion and suggestions for further research

Figure 2 presents the responsible innovation process for sustainability model as a framework to derive concrete management implications. The model builds on existing knowledge about the management of innovation processes for sustainability as a baseline to contribute to socio-technical system creation. The presented responsible innovation process for sustainability however integrates the findings from the SI and RRI literature about key management practices and how responsibility can become a strategic part of it. Thereby, the model is seen as a baseline for deriving responsible management implications to contribute to sustainable socio-technical systems. It shows that the individual process elements influence each other. The process model is centered around the RRI principles. They affect the individual innovation process phases but also are affected by the individual phases through internal attributes (management and human resources) as outlined in Section Interpretation of the results in an innovation process for sustainability to derive concrete implications for responsible management. Additionally, the principles of anticipation, reflexivity and responsiveness influence the way in which the business ideas are validated, how the network is created and to what extend the market needs are considered. The principle of inclusion influences the entire process. In the model inclusion is placed on the edge of the process, because the inclusiveness principle, unlike the others, is an essential starting condition that empowers the other principles to be implemented responsibly. Even though certain elements can be assigned to specific process phases, it is more important to see the relationships between the process phases. The emphasis on the existing interlinkages between the process phases and how they can influence each other is one of the major differences between the responsible innovation process model for sustainability and the one presented by Keskin et al. (2013). The network, for instance, becomes most evident in the design phase, the establishment of the network, however, already begins in the idea phase. This is where the cornerstones for a high-quality network are set. Therefore, responsible network building starts before the network is actually established by thinking about the most strategic partners along the value chain in order to influence socio-technical systems in a sustainable way. These interlinkages are also evident between external validation and market-orientation. Responsible market-orientation depends to a large extent on external interests and if the innovations can meet these various interests. External validation helps to grasp and raise interest early on. Subsequently, these interests should be preserved and considered throughout the whole innovation process. The emphasis on interlinkages together with the provision of tools to express them additionally contributes to strategic opportunities for moving away from linear process patterns and a dominance of the supply-side (Inigo and Blok, 2019). The connection between anticipation and responsiveness particularly shows how bi-directional learning loops can be implemented to translate anticipated knowledge into current research and innovation activities (Rose et al., 2021; Zscheischler et al., 2022). This contributes to the overall dynamic capabilities of innovation processes. By adding inclusiveness, such as including different regional perspectives into anticipatory activities, the innovation process can be further steered into a socially desirable direction (Fitjar et al., 2019; Jakobsen et al., 2019). Furthermore, by taking possible contextual conflicts into account, responsible management of sustainability trade-offs can be enhanced.

Finally, an analytical framework can be adapted, which aims to embed responsible management in innovation processes for sustainability (see Table 6).

**Table 6:** Questionnaire for responsible management in innovation processes for sustainability.

**Management and human resources**		
**Responsible management advice**	**Questions for orientation**	**References**
Responsibility experts	What knowledge/skill is needed to manage sustainability issues in a responsible way?	Ligardo-Herrera et al., 2018; Brier et al., 2020; Emilsson et al., 2020; Ketzer et al., 2020
Responsibility as integral part of the organization	What are our motivations and how can we include responsibility (e.g., moral aspects) into our value system?	
The management acts as a responsibility coordinator and provides guidelines	What responsibility standards can be used for the continual process?	
**External validation**		
Consideration of innovations and its consequences	What are the impacts of the innovation?	Keskin et al., 2013; Khan et al., 2016; Auer and Jarmai, 2018; Björklund and Forslund, 2018
Context specific inclusion e.g., in terms of knowledge (local experts)	Who are the effected people?	
Multi-disciplinary teams inside and outside	What further development opportunities are there for employees and are there specific external experts?	
Broad infrastructure perspective	Are there any infrastructure elements we are likely to oversee?	
Testing of innovations in competitive surroundings while considering responsibility aspects as a success factor	How can we go beyond current sustainability standards in order to gain competitive advantage?	
**Network**		
Establishing high-quality networks	What kind of support is needed for the individual innovation to develop?	Hallstedt et al., 2013; Kennedy et al., 2017; Pakura, 2020
Networks along the value chain (broad infrastructure)	How does the infrastructure along the value chain look like and who are the critical partners?	
Anticipation as a tool for strategic network creation today and in the future	Who are the critical auteurs today and in the future?	
**Market-orientation**		
Re-integration of generated knowledge into the innovation process	How can we establish routines to increase adaptability and responsive action?	Capurro et al., 2015; Gurzawska et al., 2017; Kennedy et al., 2017; Sonck et al., 2020
Target oriented inclusion for high-quality input	How can inclusion processes be designed in order to be target oriented?	
**Combining intended and created value**		
Definition of sustainability goals that include responsibility aspects	What are our sustainability goals and how can we cope with sustainability issues in a responsible manner?	Hallstedt et al., 2013; Timmermans et al., 2017; Ligardo-Herrera et al., 2018; Keskin et al., 2020
Transformation of “process responsibility” into “design responsibility”	Are our responsibility goals reflected in the business case and are they are core element of decision-making?	
Building up routines	What information is needed in order to inform follow-up innovation processes?	
Comparison of created values with intended values	Do the outcomes (e.g., final outcomes or intermediate outcomes) reflect the defined goals and, if not, what can we do better the next time?	

The presented questionnaire for responsible management is considered one of the central results of the analysis. The analysis has emerged from the motivation to broaden the scope of application of innovation processes for sustainability through RRI knowledge, in which social desirability is added as a main determinant in the creation of socio-technical systems. This is achieved by using the questionnaire as a guide for embedding responsible management practices into innovation processes for sustainability to generate socially desirable outcomes. The results show that the management practices proposed in the SI literature to promote socio-technical system change can be supported by the RRI principles. Consequently, they ether support or strengthen existing management practices. The findings provide first indications how innovation can be managed in such a way that it contributes to the creation of sustainable socio-technical systems. Thereby, the findings speak in favor of the assumption that if, responsibility becomes embedded in innovation processes for sustainability and, secondly, if innovation processes for sustainability can realize their abilities to influence socio-technical systems, then the creation of socio-technical systems can become generally more sustainable, as the risk of overlooking important aspects of sustainability will be reduced.

Additionally, the findings from the study allow to make suggestions for further research. First, it is suggested to deepen the knowledge on how the RRI principles can assist in the assessment of socio-technical systems in order to favor sustainable socio-technical system change in practice. The present study does not allow any conclusions to be drawn about the mechanisms that act beyond the business innovation process which could lead to changes in socio-technical systems through responsible management. However, it should be noted that there already is an existing literature that speaks in favor of the effectiveness of RRI tools to address this task (Schlaile et al., 2017; Werker, 2021; Scheer et al., 2022) even though the dynamic relationships are not well-analyzed yet. Second, it is recommended to focus on RRI as a design element for innovation. The responsible innovation process model for sustainability and the presented questionnaire for responsible management can inform research aiming to contribute to the measurement of RRI (Stilgoe, 2018; van de Poel, 2021).

This article also makes a conceptual contribution to the literature. By highlighting SI as an important concept for understanding organizations as socio-technical system builders and by raising the awareness for a strategic integration of responsible management during innovation processes for sustainability, the role of organizations as creator of a sustainable socio-technical system is further highlighted (Blok and Lemmens, 2015; Lubberink et al., 2017; Cuppen et al., 2019). Thereby, this study provides clear perspectives how responsibility can be integrated into business innovation process management to minimize the risk for creating partially-sustainable—irresponsible—socio-technical systems. This in an area, where arguably relatively little knowledge is existing (Inigo et al., 2017). Furthermore, the study contributes conceptually to the RRI literature. It outlines a possible approach for operationalizing RRI along the whole innovation processes and adds to discussions about responsible socio-technical system creation (Schlaile et al., 2017; Urmetzer et al., 2018; Jakobsen et al., 2019) by focusing on concrete implications for responsible management in underlying innovation processes for the transition toward sustainability.

Lastly, some limitations of the study need to be raised. It must be acknowledged that the inductive coding procedure was performed by the author. Although this was done to the best of the author's knowledge, it cannot be ruled out that others would have come to different conclusions. Nevertheless, it is assumed that the results are representative.

## Author's note

EU-Initiatives, such as the Horizon 2020 program to address grand social challenges, indicate a shift toward optimizing research and innovation to increase their impact on social sustainability in addition to environmental and economic sustainability. At the heart of this development is the attempt to reflect on current practices and to think about future developments in a forward-looking approach. The Responsible Research and Innovation (RRI) concept has attracted attention in this context. RRI questions research approaches to go beyond social acceptance and achieve socially desirable outcomes. The basic principles of RRI—inclusion, anticipation, reflexivity, and responsivity—aim to capture future strategic concerns and reflect on research activities early in the innovation process. However, there are still many open questions how RRI can be operationalized in the research and business context. Furthermore, even though RRI is referred to as a potential framework to support sustainability transitions at a socio-technical system level and despite the fact that some articles already successfully used RRI knowledge to inform sustainability transition research, concrete management implications for business innovation processes are currently lacking. This minimizes the contribution-power of business innovation processes to influence sustainable socio-technical system creation in which all aspects of sustainability are equally acknowledged. This paper aims to address these literature gaps. Therefore, it raises the question how innovation can be managed to contribute to the creation of sustainable socio-technical systems? The paper argues that the Sustainable Innovation (SI) literature and its conceptualization of the innovation process for sustainability already provides valuable information on important management practices along business innovation processes that assist in socio-technical system creation. However, an essential limitation of the current understanding is highlighted in this paper. Accordingly, the current SI literature that refers to innovation processes for sustainability indicates a non-holistic understanding of sustainability, that risks creating only partially-sustainable socio-technical systems despite a sustainability intention. In the eyes of the author this favors the creation of “irresponsible”-socio-technical systems. This means for instance, that the framework tends to provide insufficient room to address potential sustainability trade-offs, such as between environmental and social sustainability and risks to create socio-technical systems which are e.g., more environmentally-friendly but socially unjust. Nevertheless, the evidence from the empirical literature on the innovation process for sustainability supports the general opinion that the RRI and SI concepts can enrich each other in a joint analysis to identify possible complementarities. Therefore, in the run of this paper the RRI principles are suggestively integrated into the existing knowledge about innovation processes for sustainability to derive management implications for responsible management. This is achieved by a systematic review of empirical studies from the RRI and SI literature. Finally, the gained knowledge about management practices is synthesized in an existing innovation process model for sustainability in order to extend it to a responsible innovation process model for sustainability. This paper does not only provide first perspectives into how RRI can be operationalized in business innovation processes, but also how responsible management can be integrated in innovation processes for sustainability to influence the creation of sustainable socio-technical systems in order to better address grand social challenges. Hence, one answer to the question of how to manage innovation in a way that it contributes to the creation of sustainable socio-technical systems is to establish a framework for responsible management that can be applied along innovation processes for sustainability.

## Data availability statement

The raw data supporting the conclusions of this article will be made available by the authors, without undue reservation.

## Author contributions

The author confirms being the sole contributor of this work and has approved it for publication.

## Funding

Open Access Funding provided by the Freie Universität Berlin.

## Conflict of interest

The author declares that the research was conducted in the absence of any commercial or financial relationships that could be construed as a potential conflict of interest.

## Publisher's note

All claims expressed in this article are solely those of the authors and do not necessarily represent those of their affiliated organizations, or those of the publisher, the editors and the reviewers. Any product that may be evaluated in this article, or claim that may be made by its manufacturer, is not guaranteed or endorsed by the publisher.
